# Severity-Stratified Hyponatremia Is Associated with Increased Mortality and Complications in Nontraumatic Intracerebral Hemorrhage

**DOI:** 10.3390/jcm15103964

**Published:** 2026-05-21

**Authors:** Saketh Amasa, Vinit Reddy, Monique Mitchell, Kiran Sankarappan, Suad Hernandez, Khaled Taghlabi, Amir H. Faraji

**Affiliations:** 1 John Sealy School of Medicine, University of Texas Medical Branch, Galveston, TX 77550, USA; 2 Texas A&M College of Medicine, Houston, TX 77807, USA; 3 Houston Methodist Hospital, Houston, TX 77030, USA

**Keywords:** hyponatremia, intracerebral hemorrhage, mortality

## Abstract

**Introduction**: Hyponatremia is common after nontraumatic intracerebral hemorrhage (ICH) and has been associated with worse outcomes, although prior studies have been limited by smaller sample sizes and heterogeneous exposure definitions. This study evaluated the association between severity-stratified hyponatremia and mortality, survival, and complication rates following nontraumatic ICH. **Methods**: A retrospective cohort study was performed using the TriNetX database. Patients with nontraumatic ICH were stratified by serum sodium measurements obtained within 7 days of diagnosis. Two separate propensity score-matched analyses were conducted: moderate hyponatremia versus normonatremia (17,547 patients per cohort) and severe hyponatremia versus normonatremia (5010 patients per cohort). The primary outcome was 30-day mortality. Secondary outcomes included seizures, cerebral edema, hydrocephalus, external ventricular drain placement, tracheostomy, percutaneous endoscopic gastrostomy (PEG) placement, pulmonary embolism, deep vein thrombosis, ischemic stroke, and myocardial infarction. Statistical significance was set at *p* < 0.05. **Results**: Moderate hyponatremia was associated with increased 30-day mortality (17.5% vs. 13.3%; HR 1.324, 95% CI 1.255–1.398; *p* < 0.001), while severe hyponatremia demonstrated a greater increase in mortality (18.7% vs. 12.9%; HR 1.473, 95% CI 1.332–1.628; *p* < 0.001). Both cohorts had higher rates of seizures, cerebral edema, hydrocephalus, tracheostomy, PEG placement, deep vein thrombosis, and myocardial infarction compared with matched normonatremic controls. External ventricular drain placement was also more frequent in both cohorts. Pulmonary embolism increased in moderate hyponatremia but was not significantly different in severe hyponatremia. Ischemic stroke occurred less frequently in both cohorts. **Conclusions**: Moderate and severe hyponatremia were associated with increased mortality and complications in patients with nontraumatic ICH, with stronger associations observed in severe hyponatremia. These findings support serum sodium as a clinically relevant marker for risk stratification and monitoring during acute ICH care. However, causality cannot be established, and whether correction of hyponatremia improves outcomes requires prospective studies.

## 1. Introduction

Hyponatremia is the most common electrolyte disturbance in critically ill patients and is particularly prevalent in those with acute neurologic injuries [1]. In the setting of intracerebral hemorrhage (ICH), hyponatremia has been implicated in worsening cerebral edema, increased intracranial pressure, and adverse neurologic outcomes [2]. Although ICH accounts for a smaller proportion of strokes than ischemic stroke, it carries a substantially higher burden of morbidity and mortality, with 30-day fatality rates approaching 50% and fewer than one-third of survivors achieving functional independence at 90 days [3,4]. Hemorrhage location also influences prognosis and underlying etiology. Lobar hemorrhages have a more severe early clinical profile than deep subcortical hemorrhages and are more frequently associated with non-hypertensive mechanisms [5].

The importance of understanding the prognostic role of hyponatremia in ICH is underscored by its frequency and clinical impact. Several retrospective studies have suggested that hyponatremia may be associated with increased mortality and complications in patients with hemorrhagic stroke [6,7,8]. One prospective study by Shah et al. found that hyponatremia was an independent predictor of in-hospital mortality in this population [9]. However, these studies have been limited by small sample sizes, single-center design, and lack of adjustment for important confounders.

The objective of this study was to evaluate the association between hyponatremia and clinical outcomes, including mortality and neurologic complications, in a large multicenter cohort of patients with nontraumatic intracerebral hemorrhage.

## 2. Methods

### 2.1. Study Design and Setting

We conducted a retrospective propensity score-matched cohort study to evaluate the association between hyponatremia and clinical outcomes in patients with nontraumatic intracerebral hemorrhage (ICH). This study used data from the TriNetX Research Network (Cambridge, MA, USA), a global federated health research database that provides deidentified electronic health record data from approximately 142 million patients across 103 healthcare organizations, primarily academic tertiary care centers [10]. The TriNetX platform aggregates real-world clinical data, including diagnoses, procedures, laboratory values, medications, and vital status, from participating institutions. Because all data are de-identified and comply with the Health Insurance Portability and Accountability Act (HIPAA), this study was exempt from Institutional Review Board oversight. The data query was conducted on 26 January 2025. This study followed the Strengthening the Reporting of Observational Studies in Epidemiology (STROBE) guidelines for reporting observational studies.

### 2.2. Participants

#### 2.2.1. Study Population and Inclusion Criteria

Patients diagnosed with nontraumatic intracerebral hemorrhage were identified using the International Classification of Diseases, Tenth Revision, Clinical Modification (ICD-10-CM) code I61. Patients were eligible for inclusion if they (1) were at least 18 years of age at the time of ICH diagnosis; (2) received care between 25 January 2008, and 25 January 2023; (3) had at least one serum sodium measurement recorded within 7 days of ICH diagnosis; and (4) survived at least 24 h after ICH diagnosis to ensure accurate outcome assessment. The index event was defined as the earliest recorded ICH diagnosis within the study period.

#### 2.2.2. Exclusion Criteria

Patients were excluded if they (1) had hypernatremia (any sodium value >145 mmol/L) within 7 days of ICH diagnosis; (2) had insufficient follow-up data to assess primary outcomes; (3) had outcome events documented prior to the ICH diagnosis; or (4) had missing data for key propensity score matching variables.

#### 2.2.3. Cohort Definition

To address heterogeneity in the original exposure definition and improve clinical interpretability, we performed severity-stratified analyses using mutually exclusive hyponatremia cohorts based on serum sodium measurements obtained within 7 days of ICH diagnosis. In the first analysis, the moderate hyponatremia cohort included patients with at least one serum sodium value between 125 and 129 mmol/L and no sodium values <125 mmol/L. In the second analysis, the severe hyponatremia cohort included patients with at least one serum sodium value <125 mmol/L. Mild hyponatremia (130–135 mmol/L) was excluded from the revised primary analyses to reduce the inclusion of transient or borderline abnormalities. In both analyses, the reference normonatremic cohort included patients whose sodium values remained consistently between 136 and 145 mmol/L during the same 7-day window. Patients with any sodium value >145 mmol/L were excluded to avoid overlap with hypernatremia and reduce exposure misclassification. Severity thresholds were selected to distinguish clinically meaningful sodium derangements and reduce the inclusion of transient or borderline abnormalities [10].

#### 2.2.4. Variable Definitions and Data Collection

All variables were extracted from structured electronic health record fields within the TriNetX platform. Demographic variables included age (continuous), sex, self-reported race, and ethnicity. Comorbidities were defined using ICD-10-CM codes and included diabetes mellitus (E08–E13), essential hypertension (I10), nicotine dependence (F17), prior cerebral infarction (I63), alcohol-related disorders (F10), and disorders of lipoprotein metabolism (E78). Long-term use of anticoagulants or antiplatelets was identified using ICD-10-CM code Z79.0. Body mass index (BMI) was calculated from height and weight measurements and categorized as underweight (<18.5 kg/m^2^), normal weight (18.5–24.9 kg/m^2^), overweight (25.0–29.9 kg/m^2^), obese class I (30.0–34.9 kg/m^2^), obese class II (35.0–39.9 kg/m^2^), and obese class III (≥40.0 kg/m^2^). Laboratory values were extracted from the most recent measurements within the specified time windows.

#### 2.2.5. Outcomes

##### Primary Outcome

The primary outcome was all-cause mortality at 30 days following ICH diagnosis. Mortality data in the TriNetX platform are derived from multiple sources, including electronic health records, death certificates, and social security death index data, providing high accuracy for mortality ascertainment.

##### Secondary Outcomes

Secondary outcomes were assessed within 30 days following ICH diagnosis and included: (1) neurologic complications (seizures [ICD-10-CM: R56], cerebral edema [ICD-10-CM: G93.6], and hydrocephalus [ICD-10-CM: G91]); (2) surgical interventions (craniotomy for hematoma evacuation [CPT: 61313, 61315] and external ventricular drain placement [ICD-10-PCS: 009600Z, 009630Z, 009640Z]); (3) supportive interventions (percutaneous endoscopic gastrostomy [ICD-10-PCS: 0DH63UZ, 0DH64UZ] and tracheostomy [CPT: 31600]); (4) thromboembolic complications (pulmonary embolism [ICD-10-CM: I26] and deep vein thrombosis [ICD-10-CM: I82.4]); and (5) cardiovascular complications (ischemic stroke [ICD-10-CM: I63] and myocardial infarction [ICD-10-CM: I21]). All outcome definitions were prespecified and based on established clinical coding practices.

For patients who underwent intracranial pressure (ICP) monitoring, the most recent ICP measurement within the 30-day window was analyzed using laboratory code UMLS:LNC:60956-0.

##### Propensity Score Matching

To reduce confounding by indication and balance baseline characteristics between cohorts, we performed 1:1 propensity score matching using a greedy nearest neighbor algorithm without replacement and a caliper width of 0.1 pooled standard deviations [11]. Propensity score matching was performed separately for the moderate and severe hyponatremia cohorts, each matched 1:1 to an independently derived normonatremic control cohort using the same prespecified covariates. Propensity scores were estimated using multivariable logistic regression with hyponatremia as the dependent variable. The propensity score model included the following prespecified covariates based on clinical relevance and prior literature: age (continuous), sex, race, ethnicity, diabetes mellitus, essential hypertension, nicotine dependence, prior cerebral infarction, alcohol-related disorders, disorders of lipoprotein metabolism, BMI categories, and long-term use of anticoagulants or antiplatelets. All covariates were defined as binary variables except for age, which was treated as a continuous variable.

The TriNetX platform employs randomized row ordering to mitigate bias in the nearest-neighbor matching process. Covariate balance between matched groups was assessed using standardized mean differences (SMDs), with values <0.1 considered indicative of adequate balance [12]. The quality of matching was further evaluated by examining the distribution of propensity scores before and after matching.

##### Statistical Analysis

All analyses were performed separately within each propensity score-matched cohort pair (moderate hyponatremia vs. normonatremia and severe hyponatremia vs. normonatremia). Categorical variables are presented as frequencies and percentages, and continuous variables as means with standard deviations for normally distributed data or medians with interquartile ranges for skewed data. Baseline characteristics were compared between cohorts using chi-square tests for categorical variables and Student’s *t*-tests for continuous variables.

For the primary outcome of 30-day mortality, we used Kaplan–Meier survival analysis with log-rank testing to compare survival curves between cohorts. Cox proportional hazards regression was used to estimate hazard ratios (HRs) with 95% confidence intervals (CIs). The proportional hazards assumption was tested using Schoenfeld residuals.

Secondary outcomes were analyzed using logistic regression to calculate odds ratios (ORs) with 95% CIs. For each outcome, we calculated absolute risk differences and their 95% CIs. For continuous outcomes (ICP measurements), we used Student’s *t*-tests to compare means between groups.

All statistical tests were two-sided, and a *p*-value <0.05 was considered statistically significant. No adjustments for multiple comparisons were made for secondary outcomes, as these analyses were considered exploratory.

All analyses were performed within the TriNetX Analytics platform using integrated statistical tools. The TriNetX platform has been previously validated for real-world observational cohort studies and provides robust analytical capabilities for large-scale healthcare data analysis [9].

## 3. Results

### 3.1. Study Population and Baseline Characteristics

Two separate propensity score-matched analyses were performed comparing patients with nontraumatic intracerebral hemorrhage (ICH) and severity-stratified hyponatremia to matched normonatremic controls. The first analysis included patients with moderate hyponatremia and matched normonatremic controls, with 17,547 patients in each cohort (Table 1). The second analysis included patients with severe hyponatremia and matched normonatremic controls, with 5010 patients in each cohort (Table 2).

Baseline demographic and clinical characteristics were well balanced between the groups following propensity score matching (PSM) in both analyses. In the moderate hyponatremia analysis, the mean (SD) age was 61.5 (14.3) years in the hyponatremia cohort and 62.1 (14.2) years in the normonatremia cohort, with an absolute difference of 0.6 years (*p* < 0.001). While this difference achieved statistical significance, it is unlikely to be clinically meaningful. Sex distribution was comparable, with 40.1% female in the hyponatremia group versus 40.5% in the normonatremia group. Standardized mean differences for all variables were <0.1, confirming successful propensity score matching.

In the severe hyponatremia analysis, the mean (SD) age was 61.3 (14.2) years in the hyponatremia cohort and 61.8 (14.2) years in the normonatremia cohort. Female sex was present in 41.3% and 41.5% of patients, respectively. Baseline covariates were similarly well balanced after matching, with all standardized mean differences <0.1.

### 3.2. Primary Outcome: 30-Day Mortality

At 30 days following ICH diagnosis, 3072 patients (17.5%) in the moderate hyponatremia cohort and 2340 patients (13.3%) in the matched normonatremia cohort had died. This corresponded to a significantly increased odds of mortality in patients with moderate hyponatremia (HR 1.324; 95% CI, 1.255–1.398; *p* < 0.001) (Table 3).

In the severe hyponatremia analysis, 936 patients (18.7%) in the hyponatremia cohort and 648 patients (12.9%) in the matched normonatremia cohort had died. Severe hyponatremia was associated with an even greater increase in mortality risk (HR 1.473; 95% CI, 1.332–1.628; *p* < 0.001). The progressive increase in effect size across severity categories suggests a graded association between worsening sodium derangement and 30-day mortality (Table 4).

### 3.3. Secondary Outcomes

#### 3.3.1. Neurologic Complications

Neurologic complications occurred more frequently in both hyponatremic cohorts than in matched normonatremic controls. In the moderate hyponatremia analysis, seizures developed in 10.0% versus 8.3% of patients (OR 1.231; 95% CI, 1.144–1.324; *p* < 0.001), cerebral edema in 13.7% versus 10.9% (OR 1.292; 95% CI, 1.212–1.378; *p* < 0.001), and hydrocephalus in 9.6% versus 5.3% (OR 1.907; 95% CI, 1.755–2.072; *p* < 0.001) (Table 3).

Similarly, in the severe hyponatremia analysis, seizures occurred in 11.2% versus 8.3% of patients (OR 1.397; 95% CI, 1.222–1.597; *p* < 0.001), cerebral edema in 13.5% versus 11.1% (OR 1.245; 95% CI, 1.104–1.403; *p* < 0.001), and hydrocephalus in 8.9% versus 5.0% (OR 1.862; 95% CI, 1.587–2.185; *p* < 0.001) (Table 4).

#### 3.3.2. Surgical and Supportive Interventions

Surgical and supportive interventions were more frequently required in patients with moderate hyponatremia. Craniotomy was performed in 0.8% versus 0.4% of patients (OR 1.759; 95% CI, 1.329–2.329; *p* < 0.001), external ventricular drain placement in 3.4% versus 2.3% (OR 1.532; 95% CI, 1.348–1.742; *p* < 0.001), percutaneous endoscopic gastrostomy (PEG) placement in 5.3% versus 3.3% (OR 1.653; 95% CI, 1.486–1.838; *p* < 0.001), and tracheostomy in 2.8% versus 1.5% (OR 1.916; 95% CI, 1.648–2.228; *p* < 0.001) (Table 3).

In the severe hyponatremia analysis, external ventricular drain placement (3.3% vs. 2.2%; OR 1.499; 95% CI, 1.175–1.911; *p* = 0.001), PEG placement (4.3% vs. 3.2%; OR 1.381; 95% CI, 1.121–1.701; *p* = 0.002), and tracheostomy (2.5% vs. 1.4%; OR 1.791; 95% CI, 1.333–2.407; *p* < 0.001) remained significantly increased. Craniotomy rates were similar between groups (0.6% vs. 0.6%; OR 1.034; 95% CI, 0.625–1.710; *p* = 0.898) (Table 4).

#### 3.3.3. Thromboembolic and Cardiovascular Complications

Among patients with moderate hyponatremia, pulmonary embolism occurred in 3.2% versus 2.2% of patients (OR 1.497; 95% CI, 1.312–1.709; *p* < 0.001), deep vein thrombosis in 4.7% versus 3.5% (OR 1.366; 95% CI, 1.228–1.520; *p* < 0.001), and myocardial infarction in 3.6% versus 2.8% (OR 1.302; 95% CI, 1.156–1.468; *p* < 0.001) (Table 3).

In the severe hyponatremia analysis, deep vein thrombosis (4.2% vs. 2.9%; OR 1.440; 95% CI, 1.162–1.785; *p* = 0.001) and myocardial infarction (3.4% vs. 2.5%; OR 1.367; 95% CI, 1.083–1.725; *p* = 0.008) were significantly more common, whereas pulmonary embolism was not significantly different between groups (2.5% vs. 2.0%; OR 1.244; 95% CI, 0.954–1.621; *p* = 0.106) (Table 4).

Ischemic stroke occurred less frequently in both severity groups. In the moderate hyponatremia analysis, ischemic stroke occurred in 19.1% versus 20.1% of patients (OR 0.940; 95% CI, 0.892–0.991; *p* = 0.022). In the severe hyponatremia analysis, ischemic stroke occurred in 16.2% versus 18.2% of patients (OR 0.870; 95% CI, 0.784–0.966; *p* = 0.009).

#### 3.3.4. Intracranial Pressure Monitoring

Among patients who underwent intracranial pressure (ICP) monitoring in the moderate hyponatremia analysis, mean ICP was 10.3 ± 14.7 mmHg in the hyponatremia cohort versus 15.8 ± 19.2 mmHg in the normonatremia cohort (difference, −5.5 mmHg; *p* = 0.016) (Table 5). In the severe hyponatremia analysis, mean ICP values were similar between groups: 12.1 ± 18.5 mmHg versus 13.7 ± 18.0 mmHg (*p* = 0.744) (Table 6).

## 4. Discussion

In this large, multicenter retrospective cohort study using propensity score-matched data from patients with nontraumatic intracerebral hemorrhage (ICH), we found that both moderate and severe hyponatremia within the first 7 days of diagnosis were associated with significantly worse outcomes compared with matched normonatremic controls. Specifically, moderate hyponatremia was associated with a 32% increase in the odds of 30-day mortality, whereas severe hyponatremia was associated with a 47% increase. These associations remained significant after propensity score matching for a broad set of demographic and clinical covariates. Our findings suggest that serum sodium may serve as a clinically relevant prognostic marker for adverse outcomes in ICH, although causality and therapeutic benefit cannot be established from this study design. Mild hyponatremia was excluded to prioritize clinically meaningful sodium derangements; therefore, findings may not generalize to borderline abnormalities.

The increased risk of mortality observed in the hyponatremic cohort may be partially explained by the physiologic effects of sodium imbalance on cerebral homeostasis. Hyponatremia may promote hypo-osmolality and cellular swelling, particularly within the rigid confines of the skull, where even small changes in fluid balance can result in dramatic increases in intracranial pressure (ICP) [11]. In the setting of ICH, where mass effect and edema already compromise cerebral compliance, hyponatremia may exacerbate perihematomal swelling, reduce cerebral perfusion pressure, and contribute to secondary neuronal injury [12,13]. The higher rates of cerebral edema and hydrocephalus observed across the severity-stratified analyses are consistent with these pathophysiologic processes. Furthermore, hyponatremia has been associated with blood–brain barrier tight junction dysfunction and inflammatory cytokine release, both of which can amplify brain injury after hemorrhage [14]. Seizures were also observed more frequently in patients with hyponatremia, which may relate to sodium-driven alterations in neuronal membrane potential and excitatory neurotransmission [15,16]. However, these findings may also reflect greater baseline disease severity rather than a direct causal effect of sodium derangement.

Beyond neurologic sequelae, our findings suggest that hyponatremia in ICH is associated with a broader and more resource-intensive clinical trajectory. Patients with hyponatremia demonstrated higher rates of PEG placement, tracheostomy, and external ventricular drain placement, indicating greater supportive care needs and more severe or prolonged neurological dysfunction. This may reflect both the impact of hyponatremia on neurologic recovery and its role as a surrogate marker of systemic derangement [17]. Additionally, we observed increased rates of several systemic complications, including deep vein thrombosis and myocardial infarction. Pulmonary embolism was increased in the moderate hyponatremia cohort but was not significantly different in the severe cohort. Ischemic stroke occurred slightly less frequently in both severity groups, a finding that should be interpreted cautiously and may reflect residual confounding or competing risks. While the mechanistic link between hyponatremia and thrombosis remains under investigation, proposed explanations include a hypercoagulable state associated with neutrophil extracellular traps [18]. It is also possible that hyponatremia serves as an epiphenomenon, identifying patients with more severe hemorrhagic strokes who are subsequently immobilized, systemically inflamed, and at higher thrombotic risk [17]. Regardless of etiology, these findings highlight the importance of multidisciplinary management approaches that anticipate and address systemic complications in patients with hyponatremic ICH [19].

These observations have potential clinical implications but should be interpreted with caution. First, serum sodium should be recognized as an important dynamic parameter during the acute management of ICH. Routine sodium monitoring, particularly within the first week of hospitalization, remains clinically reasonable in stroke and neurocritical care settings. Importantly, identifying the etiology of hyponatremia is critical for appropriate management. Differentiating between syndrome of inappropriate antidiuretic hormone secretion (SIADH) and cerebral salt-wasting syndrome (CSW) is essential, as they demand opposing treatment strategies [1,20,21]. However, whether correction of hyponatremia independently improves outcomes in ICH remains uncertain and cannot be determined from the present study [21]. Currently, there are no widely accepted, evidence-based guidelines for managing sodium disturbances specifically in patients with ICH. The development of standardized protocols may improve consistency of care and warrants further investigation. Moreover, integrating predictive tools or automated alerts within electronic health record systems could aid early identification and intervention for at-risk patients.

Our results corroborate and expand upon prior research. Smaller retrospective and prospective studies, including work by Shah et al., have previously reported associations between hyponatremia and increased mortality or poor neurologic outcomes in hemorrhagic stroke populations [7,8,9,22,23,24]. However, our study represents one of the largest analyses to date, with a robust matching strategy and diverse, multicenter data, enhancing the generalizability.

This study has several limitations. First, its retrospective observational design precludes causal inference and leaves room for residual confounding despite propensity score matching. Although matching improved balance across measured covariates, unmeasured or incompletely captured confounders may remain.

Second, the temporal relationship between hyponatremia and clinical deterioration cannot be established. In patients with severe intracerebral hemorrhage, cerebral edema, fluid shifts, and neuroendocrine disturbances (e.g., syndrome of inappropriate antidiuretic hormone secretion or cerebral salt wasting) may develop early in the disease course and contribute to subsequent hyponatremia.

Third, important markers of intracerebral hemorrhage severity, including hematoma volume, Glasgow Coma Scale, hemorrhage location, intraventricular extension, perihematomal edema burden, need for mechanical ventilation, osmotherapy, and urgency of neurosurgical intervention, were not available in the database and may have contributed to residual confounding. Residual confounding related to baseline hemorrhage severity remains an important consideration, as these factors may independently influence both the development of hyponatremia and subsequent clinical outcomes. The exploratory intracranial pressure analysis was additionally limited by small subgroup sizes, incomplete monitoring availability, and exclusion of invalid measurements and therefore should be interpreted cautiously. Cause-specific mortality (neurologic vs. non-neurologic) was not available in the database. Intraventricular extension is a known predictor of poor outcome after ICH and was unavailable as a structured variable in this dataset [25].

Fourth, we were unable to assess the timing of hyponatremia onset, duration of sodium abnormalities, nadir sodium trajectory, etiology of hyponatremia, or the methods and rate of sodium correction, all of which may influence outcomes in neurocritical care populations.

Fifth, the use of ICD-coded diagnoses, procedure codes, and aggregate electronic health record data introduces the possibility of misclassification and variable coding practices across participating institutions.

Finally, the severity-stratified analyses were performed to improve clinical interpretability and reduce heterogeneity in the original exposure definition; however, serum sodium remains a dynamic exposure that cannot be fully characterized using categorical thresholds alone. Despite these limitations, the large sample size, multicenter design, and rigorous matching protocol strengthen confidence in the observed associations.

In conclusion, moderate and severe hyponatremia were associated with increased mortality, neurologic complications, and systemic morbidity in patients with nontraumatic ICH. Our findings support serum sodium as a clinically relevant biomarker for risk stratification and monitoring during acute ICH care. Further prospective research is warranted to determine whether targeted sodium management strategies improve outcomes and to identify optimal management protocols. We also believe that further studies incorporating more granular clinical data may help to elucidate the true effect of hyponatremia.

## Figures and Tables

**Table 1:** Baseline characteristics of study participants after propensity score matching.

Characteristic	Hyponatremia (*n* = 17,547)	Normonatremia (*n* = 17,547)	*p* Value	Standardized Mean Difference
**Demographics**				
Age, mean (SD), y	61.5 (14.3)	62.1 (14.2)	<0.001	0.043
Female sex	7034 (40.1)	7108 (40.5)	0.421	0.009
**Race and ethnicity**				
White	10,771 (61.4)	11,028 (62.8)	0.005	0.030
Black or African American	2722 (15.5)	2696 (15.4)	0.701	0.004
Hispanic or Latino	1177 (6.7)	1060 (6.0)	0.011	0.027
Asian	1406 (8.0)	1346 (7.7)	0.233	0.013
**Medical history**				
Diabetes mellitus	5452 (31.1)	5515 (31.4)	0.468	0.008
Essential hypertension	11,084 (63.2)	11,337 (64.6)	0.005	0.030
Disorders of lipoprotein metabolism	7136 (40.7)	7359 (41.9)	0.016	0.026
Nicotine dependence	3847 (21.9)	3928 (22.4)	0.298	0.011
Long-term anticoagulant useᵇ	2789 (15.9)	2835 (16.2)	0.503	0.007
Prior cerebral infarction	4614 (26.3)	4235 (24.1)	<0.001	0.050
Alcohol-related disorders	2470 (14.1)	2434 (13.9)	0.579	0.006
**Body mass index**				
BMI, mean (SD), kg/m^2^	27.1 (6.7)	27.3 (6.6)	0.033	0.028
Underweight (<18.5)	1464 (8.3)	1438 (8.2)	0.614	0.005
Normal weight (18.5–24.9)	5988 (34.1)	6132 (34.9)	0.106	0.017
Overweight (25.0–29.9)	6419 (36.6)	6544 (37.3)	0.167	0.015
Obese class I (30.0–34.9)	4164 (23.7)	4282 (24.4)	0.141	0.016
Obese class II (35.0–39.9)	2042 (11.6)	2092 (11.9)	0.408	0.009
Obese class III (≥40.0)	1256 (7.2)	1285 (7.3)	0.550	0.006

Data are presented as No. (%) unless otherwise indicated. All standardized mean differences were <0.10, confirming adequate balance after matching.

**Table 2:** Baseline characteristics of study participants after propensity score matching.

**Characteristic**	**Severe Hyponatremia (*n* = 5010)**	**Normonatremia (*n* = 5010)**	***p*** **Value**	**Standardized Mean Difference**
**Demographics**				
Age, mean (SD), y	61.3 (14.2)	61.8 (14.2)	0.095	0.033
Female sex	2070 (41.3)	2078 (41.5)	0.871	0.003
**Race and ethnicity**				
White	2995 (59.8)	3055 (61.0)	0.220	0.024
Black or African American	620 (12.4)	592 (11.8)	0.391	0.017
Hispanic or Latino	314 (6.3)	324 (6.5)	0.682	0.008
Asian	520 (10.4)	522 (10.4)	0.948	0.001
**Medical history**				
Diabetes mellitus	1408 (28.1)	1418 (28.3)	0.824	0.004
Essential hypertension	3123 (62.3)	3188 (63.6)	0.179	0.027
Disorders of lipoprotein metabolism	1864 (37.2)	1897 (37.9)	0.496	0.014
Nicotine dependence	1033 (20.6)	1046 (20.9)	0.749	0.006
Long-term anticoagulant use	725 (14.5)	709 (14.2)	0.648	0.009
Prior cerebral infarction	1136 (22.7)	1013 (20.2)	0.003	0.060
Alcohol-related disorders	853 (17.0)	839 (16.7)	0.709	0.007
**Body mass index**				
BMI, mean (SD), kg/m^2^	26.3 (6.3)	26.7 (6.3)	0.012	0.063
Underweight (<18.5)	482 (9.6)	443 (8.8)	0.178	0.027
Normal weight (18.5–24.9)	1777 (35.5)	1812 (36.2)	0.466	0.015
Overweight (25.0–29.9)	1772 (35.4)	1804 (36.0)	0.505	0.013
Obese class I (30.0–34.9)	1054 (21.0)	1057 (21.1)	0.941	0.001
Obese class II (35.0–39.9)	507 (10.1)	492 (9.8)	0.617	0.010
Obese class III (≥40.0)	300 (6.0)	310 (6.2)	0.676	0.008

Data are presented as No. (%) unless otherwise indicated. All standardized mean differences were <0.10, confirming adequate balance after matching.

**Table 3:** Primary and secondary outcomes at 30 days.

Outcome	Hyponatremia (*n* = 17,547)	Normonatremia (*n* = 17,547)	Odds Ratio (95% CI)	*p* Value
**Primary outcome**				
Death	3072 (17.5)	2340 (13.3)	1.324 * (1.255–1.398)	<0.001
**Secondary outcomes**				
Seizures	1763 (10.0)	1460 (8.3)	1.231 (1.144–1.324)	<0.001
Cerebral edema	2402 (13.7)	1918 (10.9)	1.292 (1.212–1.378)	<0.001
Hydrocephalus	1680 (9.6)	923 (5.3)	1.907 (1.755–2.072)	<0.001
Craniotomy	135 (0.8)	77 (0.4)	1.759 (1.329–2.329)	<0.001
External ventricular drain	604 (3.4)	399 (2.3)	1.532 (1.348–1.742)	<0.001
Percutaneous endoscopic gastrostomy	932 (5.3)	576 (3.3)	1.653 (1.486–1.838)	<0.001
Tracheostomy	499 (2.8)	264 (1.5)	1.916 (1.648–2.228)	<0.001
Pulmonary embolism	560 (3.2)	378 (2.2)	1.497 (1.312–1.709)	<0.001
Deep vein thrombosis	823 (4.7)	610 (3.5)	1.366 (1.228–1.520)	<0.001
Ischemic stroke	3355 (19.1)	3525 (20.1)	0.940 (0.892–0.991)	0.022
Myocardial infarction	638 (3.6)	494 (2.8)	1.302 (1.156–1.468)	<0.001

Data are presented as No. (%) unless otherwise indicated. All outcomes were analyzed using logistic regression. * Hazard ratio from Kaplan–Meier survival analysis.

**Table 4:** Primary and secondary outcomes at 30 days.

Outcome	Severe Hyponatremia (*n* = 5010)	Normonatremia (*n* = 5010)	Odds Ratio (95% CI)	*p* Value
**Primary outcome**				
Death	936 (18.7)	648 (12.9)	1.473 * (1.332–1.628)	<0.001
**Secondary outcomes**				
Seizures	560 (11.2)	414 (8.3)	1.397 (1.222–1.597)	<0.001
Cerebral edema	675 (13.5)	557 (11.1)	1.245 (1.104–1.403)	<0.001
Hydrocephalus	448 (8.9)	251 (5.0)	1.862 (1.587–2.185)	<0.001
Craniotomy	31 (0.6)	30 (0.6)	1.034 (0.625–1.710)	0.898
External ventricular drain	166 (3.3)	112 (2.2)	1.499 (1.175–1.911)	0.001
Percutaneous endoscopic gastrostomy	217 (4.3)	159 (3.2)	1.381 (1.121–1.701)	0.002
Tracheostomy	124 (2.5)	70 (1.4)	1.791 (1.333–2.407)	<0.001
Pulmonary embolism	125 (2.5)	101 (2.0)	1.244 (0.954–1.621)	0.106
Deep vein thrombosis	209 (4.2)	147 (2.9)	1.440 (1.162–1.785)	0.001
Ischemic stroke	812 (16.2)	911 (18.2)	0.870 (0.784–0.966)	0.009
Myocardial infarction	172 (3.4)	127 (2.5)	1.367 (1.083–1.725)	0.008

Data are presented as No. (%) unless otherwise indicated. All outcomes were analyzed using logistic regression. * Hazard ratio from Kaplan–Meier survival analysis.

**Table 5:** Intracranial pressure monitoring results.

Parameter	Hyponatremia	Normonatremia	Difference (95% CI)	*p* Value
Patients with ICP monitoring, No.	149	82	—	—
Mean ICP (SD), mm Hg ᵃ	10.3 (14.7)	15.8 (19.2)	−5.5	0.016

ICP indicates intracranial pressure. ᵃ For the hyponatremia cohort, 611 data points for 46 patients were excluded due to invalid values; 12 had no remaining labs. For the normonatremia cohort, 5520 data points for 481 patients were excluded; 77 had no remaining labs.

**Table 6:** Intracranial pressure monitoring results in the severe hyponatremia cohort.

Parameter	Severe Hyponatremia	Normonatremia	Difference (95% CI)	*p* Value
Patients with ICP monitoring, No.	35	24	—	—
Mean ICP (SD), mm Hg ᵃ	12.1 (18.5)	13.7 (18.0)	−1.59	0.744

ICP indicates intracranial pressure. **ᵃ** For the severe hyponatremia cohort, 198 data points for 15 patients were excluded because they were invalid values or outside the sanitization limit; 10 had no remaining labs. For the normonatremia cohort, 5520 data points for 481 patients were excluded; 77 had no remaining labs.

## Data Availability

The data that support the findings of this study are available from the TriNetX research network but are not publicly available due to data use agreements with participating healthcare organizations. Access to the data may be obtained through a formal request to TriNetX, subject to institutional approval and applicable data use agreements.
